# Agricultural Solid Waste as Source of Supplementary Cementitious Materials in Developing Countries

**DOI:** 10.3390/ma12071112

**Published:** 2019-04-03

**Authors:** Suvash Chandra Paul, Peter B.K. Mbewe, Sih Ying Kong, Branko Šavija

**Affiliations:** 1 Civil Engineering, Monash University Malaysia, Bandar Sunway 47500, Malaysia; suvash.chandra@monash.edu (S.C.P.); Kong.Sih.Ying@monash.edu (S.Y.K.); 2 Department of Civil Engineering, The Malawi Polytechnic, University of Malawi, Private Bag 303, Blantyre, Malawi; pmbewe@poly.ac.mw; 3 Microlab, Faculty of Civil Engineering and Geosciences, Delft University of Technology, 2628CN Delft, The Netherlands

**Keywords:** agricultural waste, oil palm ash, rice husk ash, sugarcane bagasse ash, bamboo leaf ash

## Abstract

Concrete production utilizes cement as its major ingredient. Cement production is an important consumer of natural resources and energy. Furthermore, the cement industry is a significant CO_2_ producer. To reduce the environmental impact of concrete production, supplementary cementitious materials such as fly ash, blast furnace slag, and silica fume are commonly used as (partial) cement replacement materials. However, these materials are industrial by-products and their availability is expected to decrease in the future due to, e.g., closing of coal power plants. In addition, these materials are not available everywhere, for example, in developing countries. In these countries, industrial and agricultural wastes with pozzolanic behavior offer opportunities for use in concrete production. This paper summarizes the engineering properties of concrete produced using widespread agricultural wastes such as palm oil fuel ash, rice husk ash, sugarcane bagasse ash, and bamboo leaf ash. Research on cement replacement containing agricultural wastes has shown that there is great potential for their utilization as partial replacement for cement and aggregates in concrete production. When properly designed, concretes containing these wastes have similar or slightly better mechanical and durability properties compared to ordinary Portland cement (OPC) concrete. Thus, successful use of these wastes in concrete offers novel sustainable materials and contributes to greener construction as it reduces the amount of waste, while also minimizing the use of virgin raw materials for cement production. This paper will help the concrete industry choose relevant waste products and their optimum content for concrete production. Furthermore, this study identifies research gaps which may help researchers in further studying concrete based on agricultural waste materials.

## 1. Introduction

Worldwide, increasing amounts of waste generated are a major concern for a sustainable environment. According to a report by the World Bank, ~2.01 billion tons of solid waste was generated in cities worldwide in 2016 alone, amounting to a footprint of 0.74 kg per person per day [1,2]. With rapid population growth and urbanization, annual waste generation is expected to increase by 70% from the 2016 levels to 3.40 billion tons in 2050 [1]. More specifically, there is more rapid waste generation among the urban poor regions within the most developing countries due to unsustainable waste management compared to most developed countries. Over 90% of the waste in low-income countries is either disposed in an unregulated way or openly burned. These practices have serious health, safety, and environmental consequences. Poorly managed waste serves as a breeding ground for disease vectors, contributes to global climate change through methane generation, and can promote urban violence [1]. Therefore, managing waste properly is crucial in building sustainable and livable cities. Traditional waste management is expensive and requires additional municipal budgets, making it difficult to implement in many developing countries and cities. It is thus important to develop an integrated waste management system that is sustainable, efficient, cost-effective, and socially inclusive if developing countries and cities are to manage waste sustainably.

**Figure 1:**
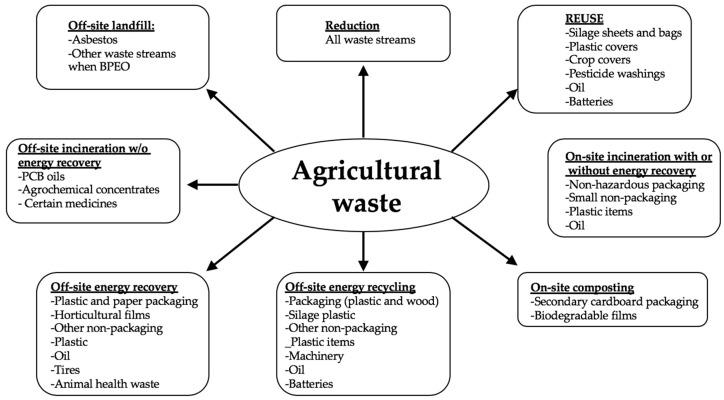
An overview of different options for agricultural waste management (after [4]).

**Figure 2:**
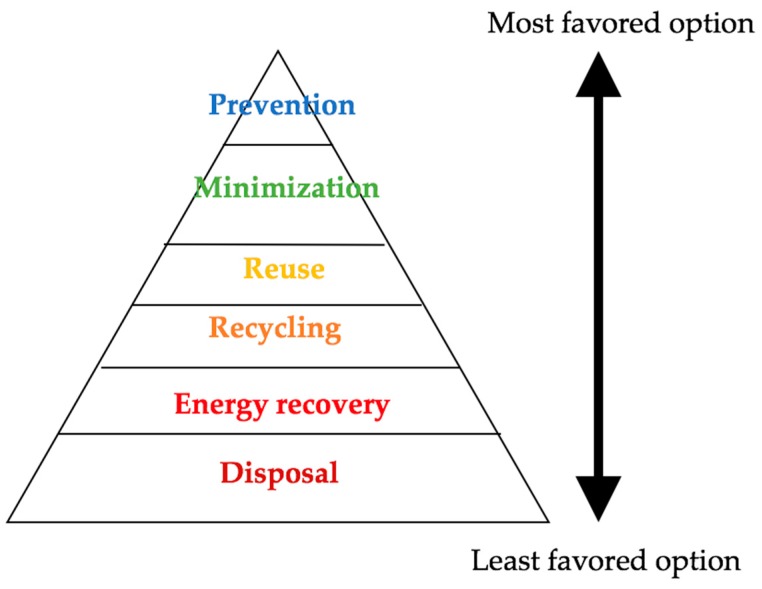
Least to most favored options for any agricultural waste (after [5]).

Typically, the excesses from growing and processing of raw agricultural products such as fruits, vegetables, meat, poultry, dairy products, and crops are classified as agricultural waste [3]. These wastes are the residues from manufacturing and processing of agricultural products and may contain materials that can benefit humans, although their economic value may be lower than the cost of collection, transportation, and processing. The composition of these wastes depends on the system and type of agricultural activities involved. Various strategies have been developed and can be employed to effectively manage agricultural wastes. An overview of different ecological and engineering options for agricultural waste management is illustrated in Figure 1 [4]. Various agricultural wastes may require unique strategies in effectively managing them. Nonetheless, ideal strategies should aim at waste prevention and minimization, as indicated in Figure 2 [5]. A lack of data related to the waste characteristics for different terrestrial regions is the major constraint in developing a comprehensive strategy for managing the agricultural waste. Hence, international database of the composition and characteristics of different agricultural waste is required [4].

One of the options for using agricultural waste is in concrete production [6,7,8]. It is well known that cement industry is a significant generator of greenhouse gas emissions [9,10,11]. Since cement is the most polluting ingredient of concrete, its use is commonly reduced through partial replacement by industrial by products such as fly ash, blast furnace slag, and silica fume [12,13,14]. However, the amount of these industrial by products is expected to reduce in the future due to, e.g., the closing of coal power plants. Furthermore, these materials are not available everywhere, and, e.g., developing countries, need to rely on different sources of “green” pozzolanic materials. It is known that processed/incinerated waste can be used as (partial) cement replacement if its composition is such that it contains sufficient quantities and ratio of CaO and SiO_2_ [15]. Research has shown that certain wastes available in developing countries possess such properties. Therefore, this review provides state of art literature on the use of different agricultural solid waste such as palm oil fuel ash (POFA), rice husk ash (RHA), sugarcane bagasse ash (SCBA), and bamboo leaf ash (BLA) in concrete production. It provides a systematic evaluation of the properties of selected agricultural wastes with regards to their intended use as cement replacement or aggregate replacement and general production processes to make them ideal for use and some challenges towards promotion of their use in concrete production. It also provides possible solutions that can be implemented after further studies/research is undertaken. The information provided in this paper should help researchers to widen their perspective about the suitability of various agricultural wastes and their influence in the production of a sustainable and greener concrete. It also highlights the research gaps with regards to the promotion of agricultural wastes in concrete production for future studies.

It should be noted that agricultural wastes can successfully be used in combination with binders other than ordinary Portland cement, such as lime and MgO cement. For example, Stevulova et al. [16] developed a biocomposite comprising 40% hemp hurds, 29% MgO cement, and 31% water. Other examples include using rice husk ash and flax for creating biocomposites in combination with inorganic matrices [17,18]. This is a promising field of research, but is outside the scope of the current review.

## 2. Processing of Agricultural Wastes for Concrete Production and General Properties 

This section describes the general processes involved in the conversion of agricultural wastes into usable materials for concrete production. It also provides the general properties of the selected agricultural wastes relevant for concrete production. Comparison of their properties with ordinary Portland cement and other relevant concrete constituents is also provided to highlight the likelihood of their use as cement replacement material.

### 2.1. Palm Oil Fuel Ash (POFA)

Palm oil fuel ash (POFA) is an important cash crop in several tropical countries, particularly Malaysia and Indonesia [19,20]. The palm oil industry produces considerable amounts of waste in the form of fibers, shells, and empty bunches discharged from the mills [19]. For every 100 tons of fresh fruit bunches processed, there will be approximately 20 tons of nut shells, 7 tons of fibers, and 25 tons of empty bunches discharged from the mills [21]. This results in significant amounts of waste, which need to be disposed of because of pollution. Shell and fiber can be used as fuel in power plants. POFA is a byproduct of this process [21]: after incineration at 800 °C to 1000 °C, ~5% ash by weight is produced [22]. This ash does not have the nutritional value to be used as fertilizer, and is commonly landfilled. 

POFA can be used in concrete either as aggregates, supplementary cementitious materials or as filler material [23,24]. Typically, POFA has high amorphous content with silicon dioxide (SiO2) as the main constituent. Table 1 and Table 2 show the chemical composition and other characteristics of POFA in Malaysia [25]. As shown in Table 1, POFA is very rich in SiO_2_ compared to cement. Due to its pozzolanic behavior, POFA has been used by researchers in cementitious materials either to replace the binder or as filler materials [23,24,26]. Table 3 shows the typical physical properties of POFA. From Table 3, it can be seen that the specific gravity, density, and water absorption of fine and coarse POFA vary from 1.7 to 2.2, 780 to 1120, and 1 to 26, respectively.

**Figure 3:**
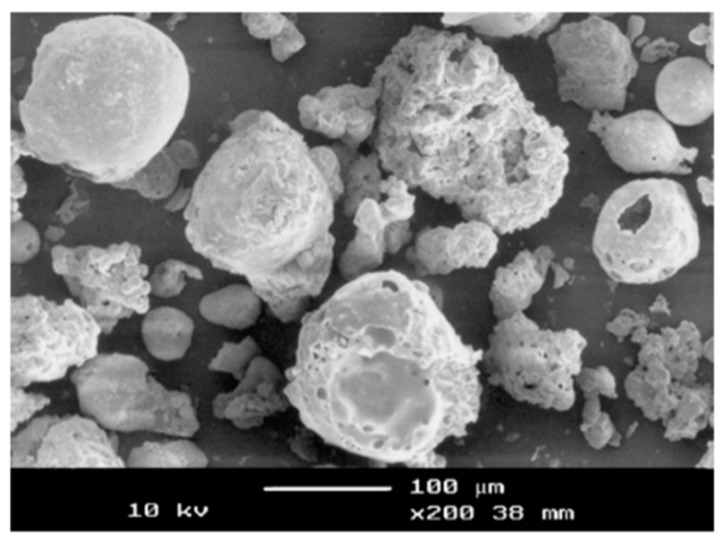
SEM micrograph of palm oil fuel ash [30].

The ash should be sieved in order to remove the organic and uncombusted matter. Furthermore, the reactivity of the ash may be increased by reducing the particle size by e.g., milling [30]. A scanning electron micrograph of POFA (before grinding) is shown in Figure 3. 

### 2.2. Rice Husk Ash (RHA)

Rice is one of the three major food crops in the world and Asian countries like China, India, Indonesia, Vietnam, and Bangladesh are the leading rice producers [31]. Rice husk is the outer covering part of the rice kernel. Being nonedible, it needs to be removed from the rice grain [31]. Although it can be used to produce various useful products such as activated carbon, sodium silicate [32,33], silicon carbide [34], or silicon nitride [35], its practical utilization is limited due to economic reasons. If unused, rice husk needs to be landfilled, where it can self-burn and create a problem for the environment [36]. 

When completely incinerating the rice husk in appropriate conditions, the residue (i.e., the rice husk ash) contains 90–96% amorphous silica (SiO_2_), which can be used as alternative binder for concrete [37]. For every 1000 kg of milled rice paddy, ~200 kg (20%) of husk is produced, which, when completely burnt, leaves ~50 kg of rice husk ash. Considering that the world production of rice paddy reached over 595 million tons in 1999 [31], this is a huge amount.

Apart from silicon dioxide, RHA contains other chemicals, as summarized in Table 4. Note that the chemical composition is dependent, to a certain extent, on the burning method, and some deviations from the given values might be present [31,38,39,40,41,42]. The main impurity of ashes is unburnt carbon, since production of carbon-free RHA requires either sophisticated equipment or very long combustion times at low temperature, which makes the burning process inefficient. Nevertheless, a recent study has reported that the carbon can be effectively eliminated by optimizing the heating rate and cooling process [43]. In this way, impurities can be removed from RHA effectively.

**Figure 4:**
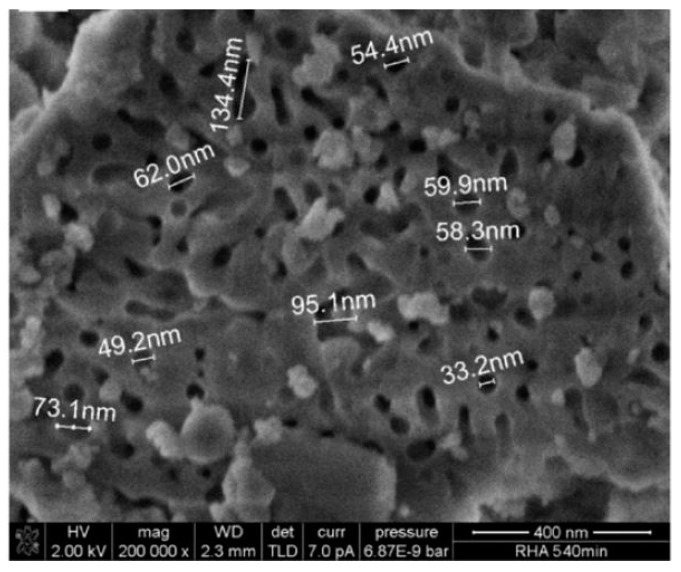
SEM images of microstructure of rice husk ash showing its porous microstructure [51].

Rice husk ash resembles silica fume in some aspects: it has a large specific surface area and a high content of amorphous silica [31]. However, they are very different in terms of particle size: while silica fume consists of very fine particles (average diameter 0.1 μm and specific surface area of 20 m^2^/g) [46], rice husk ash consists of larger particles (<75 μm in diameter) that also have high specific surface area (40–100 m^2^/g) [47,48]. This is because rice husk ash is highly porous in nature (see an electron micrograph image in Figure 4, so it has an extremely high surface area, with relatively large particle sizes. In fact, some authors suggested that, since RHA derives its pozzolanic properties from its internal surface area, grinding to a high degree of fineness should be avoided [49]. In addition, long periods of grinding increase the cost of production, especially in developing countries where electricity is costly and its supply is uncertain [50]. Accordingly, there exists an appropriate fineness of RHA which is advantageous from both technical (i.e., pozzolanic activity) and economical aspects (energy expenditure). Therefore, the successful use of RHA in concrete-like materials would benefit countries where RHA is easily accessible in both economic and environmental terms. 

### 2.3. Sugarcane Bagasse Ash

Approximately 60–70% of total sugar is produced from the sugarcane world-wide. Sugarcane production in countries such as Brazil, India, China, and Thailand is increasing every year. However, every 1000 kg of planted sugarcane produces around 231 kg (28%) of bagasse and 430 kg (52%) of solid waste [52]. The bagasse is produced as a fibrous residue after crushing and juice extraction in water media in sugar factories [53]. In the past, bagasse wastes were burned as means of solid waste disposal. In recent years, however, these residuals are used as the principal raw materials in cogeneration plants to produce electrical energy, e.g., in Brazil [54]. This results in ~2.7 million tons of ashes in Brazil alone, which are commonly accumulated in landfills [54]. Therefore, the reuse of industrially process sugarcane bagasse ash (SCBA) from bagasse can solve the dumping problem of waste generated from the sugar production process. 

The chemical composition of SCBA shows that a large proportion (> 65%) of it is SiO_2_ [55], which is almost three times higher than OPC. It also contains considerable amount of Al_2_O_3_, Fe_2_O_3_, and CaO as shown in Table 5. The presence of Al_2_O_3_, Fe_2_O_3_, and CaO in SCBA make it a good candidate as an alternative binder to cement in concrete production [35]. Other chemical and physical properties of SCBA are summarized in Table 5. From Table 5, it can be seen that the chemical compositions of SCBA are similar to the POFA. Therefore, having similar chemical properties, SCBA can also be considered as a substitute of cement in concrete. It can be used as an alternative cementitious material [23,24,25,26]. However, more research data are required to understand the chemomechanical properties of SCBA.

**Figure 5:**
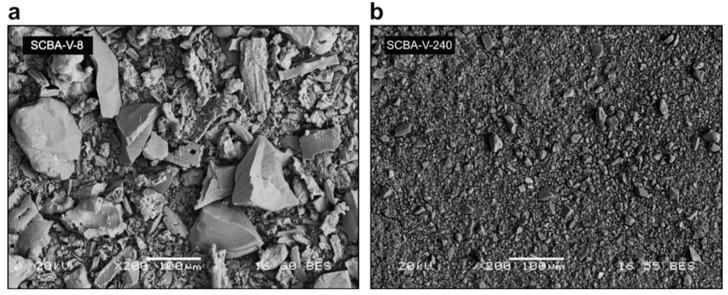
SEM images of sugarcane bagasse ashes (SCBAs) produced after 8 min (**a**) and 240 min (**b**) of vibratory grinding [57].

Similar to palm oil fuel ash, the reactivity of the sugarcane bagasse ash can be improved by reducing its particle size, e.g., by milling or grinding [57,58]. In Figure 5, scanning electron micrographs of sugar cane bagasse ash subjected to different grinding procedures are given.

### 2.4. Bamboo Leaf Ash

The annual production of bamboo is ~20 million tons, of which more than 10 million tons are produced in India, China, and Japan [59,60]. Bamboo leaf ash (BLA) is obtained by burning the dry bamboo leaves at high temperature (600 °C). Similar to RHA and SCBA, the amorphous nature of BLA is also rich in SiO_2_ (~76%), and shows high pozzolanic reaction when used as binders for producing concrete [61,62]. Other properties of BLA are summarized in Table 6. Although not a lot of research has been performed on the use of bamboo leaf ash in concrete, it is of great interest given the large amounts of wastes potentially available for cement production. The higher SiO_2_ content in BLA can expedite the reaction of calcium hydroxide crystal by forming more calcium silicate hydrate. Thus, BLA can also be an alternative binder for concrete production in developing countries and reduce their dependency on cement. 

From the above discussion it can be seen that the physical and chemical properties of POFA, RHA, SCBA, and BLA are in many ways similar to the traditional binders such cement, fly ash, and slag of cement-based materials. Therefore, it is worth doing research on these materials and scrutinize their different properties before they can be used as a supplementary cementitious material. Successful uses of these materials in concrete production may improve the basic material properties and at the same time reduce the CO_2_ negative footprint.

## 3. Agricultural Wastes as Cement or Aggregate Replacement in Concrete

Due to pozzolanic reactivity of the agricultural wastes discussed, various investigations on the behavior of the ashes generated from these wastes as cement replacements or aggregate replacements have been conducted. Favorable results have widely been obtained as discussed in this section.

### 3.1. Palm Oil Fuel Ash

**Figure 6:**
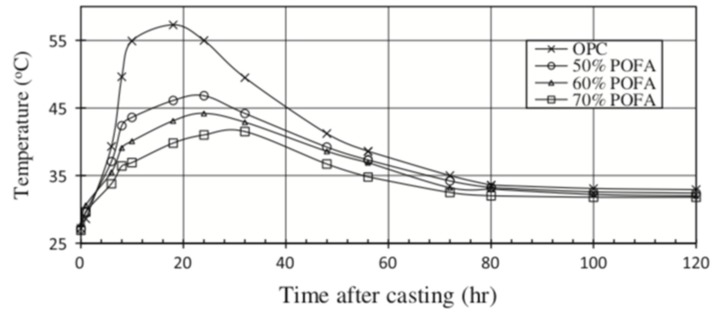
Temperature development in Portland cement concrete (OPC) and high-volume palm oil fuel ash (POFA) concrete [64].

The use of palm oil fuel ash (POFA) as partial cement replacement has a great influence on the microstructural development of concrete [22,64,65,66]. It has pozzolanic properties, and like fly ash and oil palm ash has potential to control heat of hydration of concrete (Figure 6). This is because palm oil fuel ash has low pozzolanic reactivity in the early hydration stages (i.e., first 7 days [65]). As such, palm oil fuel ash has potential to be used in massive concrete for preventing thermal cracking due to excessive heat rise.

**Figure 7:**
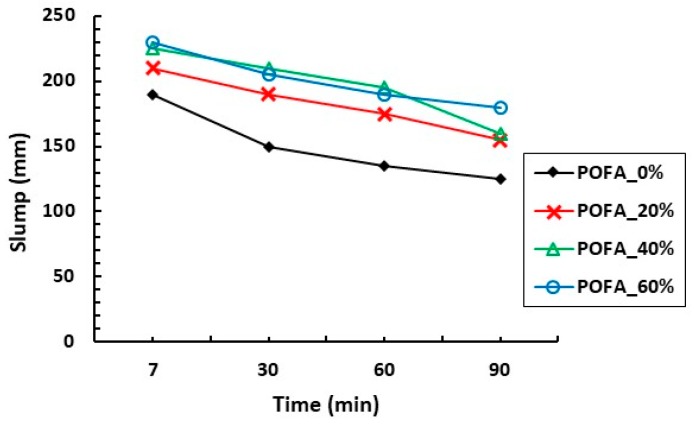
Influence of different percentages of cement replacement by POFA on concrete slump loss [20].

Utilization of POFA in concrete has an effect on its fresh properties. The influence of different percentages of ultrafine oil palm ash (max size 11 μm) replacement in concrete slump was investigated by Zeyad et al. [20], as shown in Figure 7. It can be seen that the slump increased with the increase of POFA percentage in the mix. Similar behavior was found in other studies [9,19,20]. 

A study of Tangchirapat et al. [67] showed that superplasticizer was required in the mixes in order to have similar range of slump value in control mix as well as mixes with 10%, 20%, and 30% POFA. Higher amounts of superplasticizer were required (~80% higher) for concrete with 30% OPA than for the control mix. Clearly, this is not in agreement with the results found by Zeyad et al. [20]. Tangchirapat et al. [67] used OPA with a median size of 10.1 μm after grinding the original POFA size of 65.6 μm. The morphological analysis of original POFA showed an irregular particle shape which may lead to the lower workability as the percentages of OPA increase. Therefore, higher amounts of superplasticizer are required for similar workability. From the above discussion, it is clear that the slump of concrete with POFA is dependent on various factors and still cannot be predicted a priori. The reason of this variability in the results can be attributed to the different types, source and size of POFA used by the researchers in different countries. Therefore, a comprehensive study to develop proper guidelines is required for this waste material before it can be used in concrete production.

POFA can also be used as aggregate in concrete production, having different effects on the slump. Kanadasan and Razak [26] investigated the use of waste POFA as coarse and fine aggregates in production of self-compacting concrete (SCC). In their study, 25% to 100% natural aggregates were replaced by POFA. As the replacement of POFA was increased 25% to 100% in the mixes, the slump value increased approximately 3% to 8%, compared with the reference mix. The higher slump value was reported by the less optimal particle packing of POFA in SCC. This correspond to the highest paste volume in the mix which provides a good coating and lubricating effects to the aggregates and thus increases the slump. This result is also proved by Mannan and Ganapathy [68], where the slump was more than double in 100% POFA concrete compared to the control mix. The workability of concrete is also influence by the shape of POFA. Round shape of POFA may lead to improved workability of concrete compared to other shapes [24]. However, controversial results were also reported by the researchers where slump value decreased as the percentages of POFA increased [29]. This was due to the higher water absorption of POFA compared to natural aggregates. POFA is porous and thus absorbs water from the mix, thereby reducing the water to cement (w/c) ratio. The lower slump of POFA can be improved by adding superplasticizer in the mix, as reported in [69,70].

**Figure 8:**
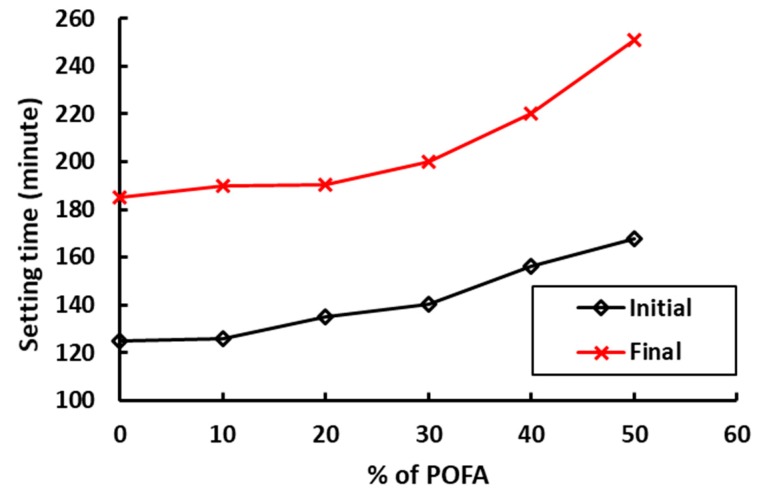
Setting times of concrete with different percentages of POFA [21].

Use of palm oil fuel ash has an impact on the initial and the final setting time of concrete (Figure 8). With increasing percentage of POFA replacement, both the initial and the final setting time increase [19,21]. The increase in setting times is not a concern, however, as both the initial and the final setting times are well within the requirements of American and British standards.

**Figure 9:**
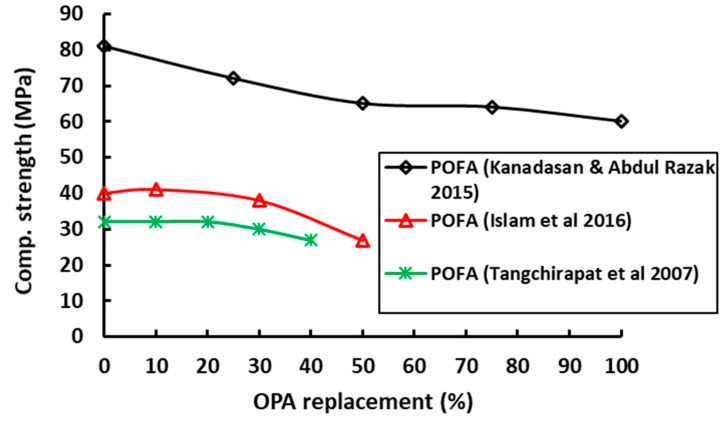
Influence of POFA on the compressive strength of concrete [26,30,71].

Studies on the compressive and tensile strengths of concrete when POFA was used show a reduction in strength with increase in the POFA content in the mix, as illustrated in Figure 9 [26,30,71]. For 20–40% cement replacement by POFA, the compressive strength of concrete at 28 days reduced to 31–66% of the control mix concrete. This trend of strength reduction was also observed in concrete with 100% of POFA. As the ratio of POFA in the mixes increased, the compressive strength of concrete gradually decreased [70]. For low replacement levels (10%), it was shown that strength at later ages (1 year) can be equal to the reference mixture [21]. In general, lower strength of POFA concrete can be attributed to the lower density of POFA as well as its porous nature. Although the concrete strength reduces with increase in POFA, the strength is still suitable for development of lightweight structural concrete [70]. Furthermore, continuous hydration of ultrafine POFA concrete cured in water resulted in higher strength compared to air curing [70].

At lower water-to-binder (w/b) ratio (e.g., 0.3), concrete made with 30% cement replacement by POFA showed a similar range of compressive strength as concrete without POFA [71]. In this case, 100% POFA was used as coarse aggregate. In a similar study [22], the authors claimed that it is possible to make structural lightweight concrete using 100% POFA, satisfying the required concrete properties. 

Long-term and durability properties of concrete are also affected by addition of palm oil fuel ash as partial cement replacement. It has been shown that POFA has a positive effect on durability: it decreases the chloride diffusivity of the mix [47,72,73], lowers water absorption [20,67,74] and water permeability [75,76,77], and increases sulfate resistance [67,78]. Furthermore, it has been identified that partial cement replacement by POFA can be used to suppress alkali silica reaction [79]. 

Although some properties of concrete (most notably strength and setting time) are adversely affected by POFA, research has shown that it can be used in many applications. As such, it can be a great resource in developing countries. Future research needs to promote its use in structural applications.

### 3.2. Rice Husk Ash

Considering that rice husk ash has a very high specific surface area, it has an important effect on the hydration of blended concrete [80]; it has a high pozzolanic reactivity [81,82,83]. As with other pozzolanic materials, calcium hydroxide is consumed by the pozzolanic reaction, leading to reduced porosity [80] and even an improved interfacial transition zone (ITZ) compared to reference concrete [84]. This causes the failure to occur through the aggregate which is intended as this phase has higher strength than the transition zone and improves the mechanical properties of the concrete including compressive, tensile and flexural strengths [85]. The addition of rice husk ash was found to increase the degree of cement hydration at later ages, which can be attributed to its internal curing ability [80]. 

Use of RHA as supplementary cementitious material affects the fresh properties of concrete. While some studies showed that the addition of rice husk ash was found to increase slump compared to reference (i.e., Portland cement) concrete [86], others found a moderate decrease in slump [87]. The effect of RHA on the setting time (initial and final) is still under debate: while some authors found that the setting times are increased in proportion to the RHA addition percentage [88], others found a moderate decrease in setting time proportional to the RHA addition percentage [89]. RHA addition has other positive contributions to the properties of fresh concrete: according to Le and Ludwig (2016) [51], bleeding and segregation of self-compacting high-performance concrete can be reduced by incorporation of RHA. The incorporation of RHA improves the physical structure of binders like cement and increases the plastic viscosity [85]. This effect is more noticeable at higher percentages of RHA content in the mix. The macromesoporous structure of RHA (see Figure 4) could induce great intermolecular attraction forces and can be used as viscosity modifying agent in concrete [24]. As a pozzolanic material, RHA reacts with calcium hydroxide during the hydration process of cement and improves the aggregate–paste connectivity [85]. This improves connection of aggregate–paste forces failure to occur in the aggregates when load is applied. Hence, the mechanical properties of concrete including compressive, flexural and tensile strength improve when RHA added.

**Figure 10:**
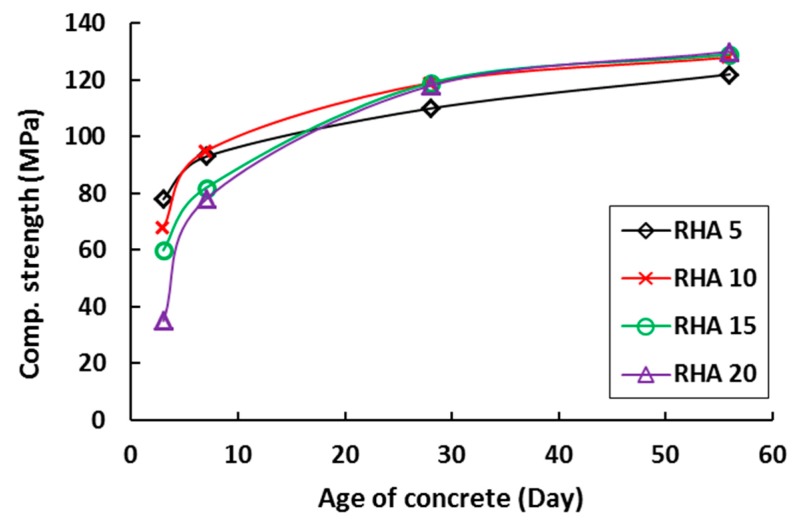
Influence of rice husk ash (RHA) on the compressive strength development of concrete [51].

The addition of RHA to concrete causes an increase in compressive strength, as shown in Figure 10. However, no noticeable difference in strength was observed with RHA content between 10 to 20%. It was also noted that 20% of RHA content in concrete increased its strength by ~20% higher than that of OPC [90]. The compressive strength development up to 365 days showed about 13% higher strength in 10% RHA concrete than in control mix of OPC [91]. This was corroborated by Hesami et al. [85], who found 14% higher strength than OPC was reported for 10% RHA replacement. Similar result was also reported by Habeeb & Mahmud [92], where maximum strength was found at 10% RHA replacement and no difference in strength was reported with 20% RHA when compared to the 10% RHA mix.

In another study [93], natural sand (average particle size 95 μm; specific gravity 2.59) was replaced with RHA (average particle size 28 μm; specific gravity 2.13) in a range of 25 to 100% to produce autoclaved aerated concrete (AAC). Inclusion of RHA negatively affected the compressive strength of AAC. Approximately 22–58% lower compressive strength was observed in AAC for 25–100% RHA content. The lower strength of AAC could be ascribed to the higher water requirement of RHA, which negatively affected the compressive strength of the AAC [93].

Durability of concrete was found to improve with incorporation of RHA as partial cement replacement. This can be attributed to the refinement of the pore structure caused by the pozzolanic activity. Concrete with RHA has shown to be more resistant to chloride ingress [72,87,94], to have lower water permeability [95] and water absorption [96], improved sulfate resistance [97,98], and lower susceptibility to alkali silica reaction [98,99]. 

Other concrete properties such as thermal conductivity and autogenous shrinkage were also investigated when RHA was incorporated in the concrete mix [36,100]. Although the thermal conductivity of the RHA insulators was found lower, it was still higher than a commercial thermal insulator made from diatomaceous silica, used as reference [36]. Similarly, RHA was found to mitigate the autogenous shrinkage of ultrahigh-performance concrete (UHPC). The mesoporous structure of RHA absorbs the free water from the mix and reduces the effective w/b and thus improving bleeding, segregation, workability, and compressive strength [100]. Furthermore, very fine (nano-)particles of RHA improved heat evolution during the early-age cement hydration. Researcher also produced nanoparticles from the agricultural waste, such as OPA, RHA, etc., and used them to improve the heat evolution during the early-age cement hydration. It was reported that nano-OPA and nano-RHA accelerate the hydration process at faster rate than the OPC [101]. These nanoparticles help rapid formation of calcium silicate hydrate (C–S–H) in the binding paste thus improve mechanical and durability properties of concrete [102,103].

### 3.3. Sugarcane Bagasse Ash

**Figure 11:**
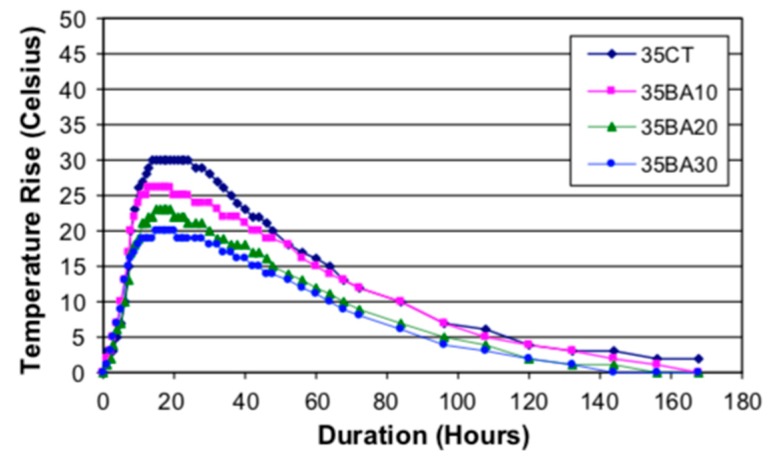
Semi-adiabatic temperature rise in concrete containing sugarcane bagasse ash (a number at the end indicates the percentage of sugarcane bagasse ash) [106].

Like the other waste materials discussed, sugarcane bagasse ash influences the hydration and microstructural development of blended concrete. Sugarcane bagasse ash has pozzolanic properties [57,104,105]. Use of sugarcane bagasse ash as partial cement replacement causes a reduction in hydration heat compared to the reference concrete [55]. The reduction of temperature rise is proportional to the percentage of sugarcane bagasse ash replacement (Figure 11). Due to such properties, sugarcane bagasse ash can be used for temperature control in mass concrete.

Workability of concrete reduces with the increase in the amount of SCBA content in the concrete mix [107]. The loss of workability is attributed to the fineness of SCBA (which is lower than cement), which absorb more water from the mix, leaving concrete drier, and consequently, less workable [108]. Other studies, however, reported an increased concrete workability with increase in the SCBA content [109]. For concrete with 25% SCBA, about 128% higher slump was reported than the control mix. The authors concluded that the addition of SCBA in concrete reduces the water demand. Another study also reported that the replacement of cement with SCBA increased the workability of concrete; therefore, no extra superplasticizer would be needed [110]. The initial and final setting time of concrete also reduced ~15–20% when 15% SCBA was replaced in concrete [108]. This lower setting time can be useful for specific applications, e.g., in concrete repair applications [111,112].

In case of mechanical properties, a study with SCBA replacement in concrete showed equal or marginally better strength than the control mix of concrete, even at early age of three days [55]. At 28 days, with 20% SCBA, a maximum 11% higher compressive strength than control mix was found. The results also indicated that up to 25% of SCBA replacement could be used in concrete production without sacrificing the strength. The optimum level of SCBA content (i.e., no strength difference from the control mix) was 20% in their study [113]. In another study, 5% and 10% of total cement content was replaced by SCBA, and then the compressive strength was compared with control mix without any SCBA [110]. It was observed that a maximum 12% higher strength was found in concrete with 5% SCBA content. With 10% SCBA content, the strength was 4% higher than the control mix. Furthermore, tensile and flexural strengths were also improved when SCBA was added to the mix. With 10% SCBA, a maximum 50% and 12% higher tensile and flexural strengths of concrete respectively than the control mix were observed. However, there was reduction in strength when SCBA content increased to 25%. At this level, similar strengths were noticed in both SCBA concrete and in control mix.

**Figure 12:**
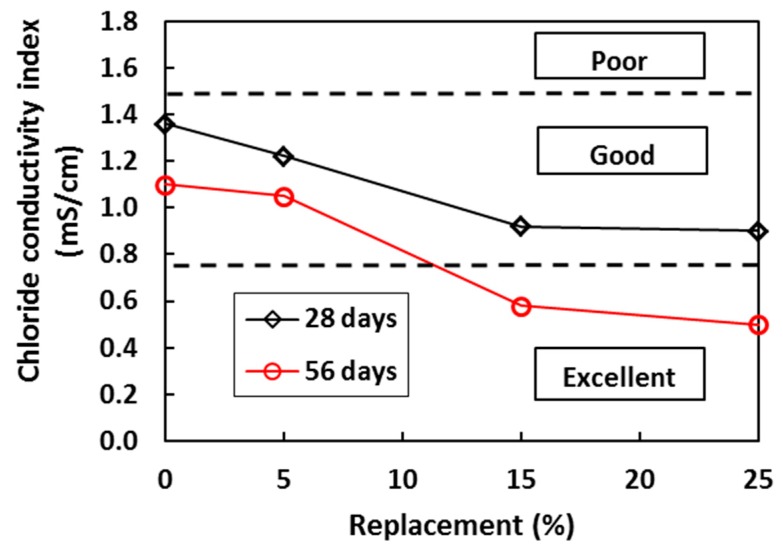
Effect of sugarcane bagasse ash on the chloride conductivity index of concrete [55].

The addition of sugarcane bagasse ash has a positive effect on concrete durability. The durability performance of concrete with different SCBA content against chloride, gas and water penetration was also investigated by the researchers [55,114]. Resistance to chloride ingress increases with the increase in SCBA content (see Figure 12). Regarding transport of water, contradictory findings have been reported: while some authors found a marginal increase in sorptivity with the increase in SCBA content [55], others reported a decrease in water permeability [106]. Some authors suggested that the sorptivity increase is due to porous nature of SCBA and the impurities in it [114]. 

From the above discussion, it can be said that the SCBA influences the quality of concrete. It has a good chemical composition and physical properties such as fineness, setting time and compressive strength. However, there is uncertainty about the optimum content of SCBA in concrete as the current results show variability in concrete strength with SCBA replacement ranging from 5% to 25%. Nevertheless, the use of SCBA in addition to the concrete is a very feasible option in improving the mechanical properties of the concrete, besides providing a suitable destination to agroindustrial by-product [107].

### 3.4. Bamboo Leaf Ash

**Figure 13:**
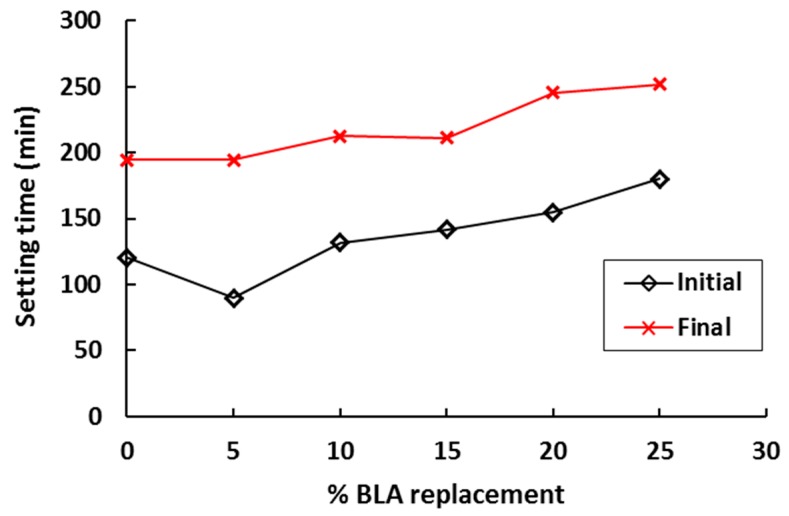
Influence of bamboo leaf ash on the setting time of concrete [115].

The use of bamboo leaf ash in concrete is less studied compared to other agricultural wastes discussed. Singh et al. [61] investigated the hydration of bamboo leaf ash blended Portland cement. They found that replacement of cement with 10% BLA delayed the initial and final setting of concrete by approximately 29% and 37% compared to the control mix. At 20% BLA replacement, these setting times reduced and were equal to those of the control mix. However, in another study, it was found that the setting of concrete was delayed as the percentages of BLA content increased as shown in Figure 13. The maximum reached 49% and 30% higher initial and final setting times of concrete were reported with 25% BLA [115]. The pozzolanic activity of BLA increased with the increase in time and temperature.

In terms of concrete workability, the higher the percentages of BLA replacement, the lower the slump was observed [116]. At a given w/b, the lower percentages of BLA (10–20%) showed improvement in workability by reducing bleeding and segregation. However, when BLA replacement was 30–40%, the concrete was unworkable. The reason was the cellular bamboo leaf ash particles and the higher fineness of BLA compared to Portland cement. The reduced workability was also observed in the study by Singh et al. [61]. Therefore, in order to improve the workability and consistency of the mix, a proper dosage of additional water or superplasticizer are required.

**Figure 14:**
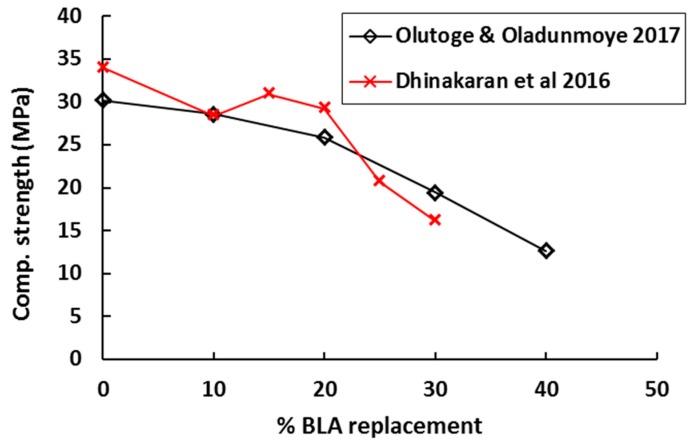
Influence of bamboo leaf ash replacement on the setting time of concrete [116,117].

When the mechanical properties of BLA concrete are concerned, the majority of studies reported that the strength of concrete reduces with increase in the BLA content [59,116,117]. As shown in Figure 14, the compressive strength of concrete gradually reduces as the amount of BLA replacement increases. However, for high-strength mortars, the strength of mortar with BLA replacement was similar to that of the control mix at 28 days. The mixes with BLA had high water demand [118]. At 10% and 20% BLA replacement in concrete, the physical and mechanical property requirements for concrete were in accordance with EN 197-1 standard [118]. In another study, the optimum replacement of OPC with BLA for the selected grade was found to be 15%. At 28 days, an ~10% reduction in the strength was observed with BLA, which was found acceptable by the authors [118].

Concrete durability properties such as acid resistance and chloride resistance are considerably improved at 10% replacement of cement with BLA [59]. The capillary suction test of 15% BLA concrete showed that the sorption coefficient was less than that of the control concrete. It was concluded that the presence of 15% BLA in the mix improves the durability of concrete by filling the voids in the cement [117]. Therefore, from durability point of view 10–15% BLA replacement with cement in concrete can be considered beneficial. Clearly, more studies are needed on BLA concrete.

## 4. Challenges for Use of Agricultural Waste in Concrete Production

Agricultural waste is widely available in developing countries, and its use as fuel is attractive from an economic point of view. The ashes produced from the fueling process have little industrial use. However, if these wastes are burnt under controlled temperature and time, they are proven to have pozzolanic properties [119]. This enables their use as supplementary cementitious materials in concrete production, which is beneficial especially in locations where other supplementary cementitious materials (SCM’s) are scarce. Nevertheless, current understanding of the hydration mechanism of these materials and the parameters influencing is incomplete. In addition, the long-term performance of these materials in concrete is yet to be proven.

One of the major issues with the agricultural wastes is that often these need to be treated such as burning at high temperature or grinding for having the desire shape and size. However, this process requires lot of energy and also contributes to the CO_2_ emission. Therefore, research needs to concentrate to find out the actual benefit of reusing these waste materials in concrete. This is in addition to potential technical benefits, the use of agricultural waste as alternative binder can lower production cost of concrete, lower cement consumption and thus CO_2_ emission [90].

So far, limited research has focused on the production and processing costs of these waste materials [119]. In general, these wastes must be treated from their original/source condition to the stage where these are ready to use in concrete as partial replacement of binders. All of this information is needed in order to be able to assess the lifecycle impact of these (potential) SCMs and possibly improve their production methods where possible. This is critical, as lower overall lifecycle costs would provide a boost for research and application of these materials in engineering practice in developing countries.

## 5. Summary, Conclusions, and Recommendations

This article provides an overall summary of research devoted to utilization of various agricultural wastes in concrete production, especially in developing countries. For the various waste sources discussed, it was found that they can be used as partial cement replacements or aggregate materials in concrete. If properly treated, the ashes have pozzolanic properties and contribute to the hydration and hardening of concrete. If properly designed, such concretes are not inferior to OPC concretes and, in fact, they can show improved workability and long-term durability, together with reduced heat of hydration. This makes such wastes suitable candidates for various specific applications, e.g., for creating durable concrete or massive concrete structures.

At the moment, it can be considered that the feasibility of use of agricultural waste materials in concrete has been proven. Although more studies need to focus especially on long-term properties, progress is needed first and foremost in production technology and quality control of these materials. Only when ashes of constant high quality can be produced at low financial and environmental costs will their use in engineering practice become more widespread. This is important, as for many developing countries these are the only SCMs available. 

## Figures and Tables

**Table 1:** Comparison of chemical compositions of Portland cement and palm oil fuel ash [25].

Major Chemical Composition %	Cement	POFA
Silicon Dioxide (SiO_2_)	19.98	66.64
Aluminium Oxide (Al_2_O_3_)	5.17	3.82
Iron Oxide (Fe_2_O_3_)	3.27	3.70
Calcium Oxide (CaO)	63.17	5.23
Magnesium Oxide (MgO)	0.79	2.29
Sulfur Trioxide (SO_3_)	2.38	0.43
Loss on Ignition (LOI)	2.5	2.32

**Table 2:** Palm oil fuel ash characteristics [25].

Type	Remarks
Appearance	Brown to light brown colored liquid
Total solids (%)	40
PH solution	7.5 to 8.0
Salt content	Max. 5%
Insoluble materials	Negligible
Chlorides as NaCl	Nil

**Table 3:** Physical properties of palm oil fuel ash.

Authors	Size of POFA	Bulk Density (kg/m^3^)	Specific Gravity	Moisture Content (%)	Water Absorption (%)
Kanadasan & Razak [26]	Fine	-	1.97	0.5 ± 0.25	10 ± 5
	Coarse	-	1.73	1.0 ± 0.5	3 ± 2
Abdullahi et al. [27]	Fine	1120	1.8	-	14.3
	Coarse	790	1.7	-	5.4
Mohammed et al. [28]	Fine	1120	2.0	0.11	26.4
	Coarse	780	1.8	0.07	4.4
Ahmad et al. [29]	Fine	1040	2.2	-	-
	Coarse	860	1.8	-	4.6

**Table 4:** Typical chemical and physical properties of rice husk ash [44,45].

Chemical Composition (%) (Minor Constituents not Given)					
SiO_2_	Al_2_O_3_	Fe_2_O_3_	CaO	MgO	K_2_O
93.4	0.05	0.06	0.31	0.35	1.4
Physical properties					
Fineness—median particle size (µm)			8.6		
Specific gravity			2.05		
Pozzolanic activity index (%)			99		
Water absorption (%)			104		

**Table 5:** Typical chemical and physical properties of sugarcane bagasse ash [56].

Chemical Composition (%) (Minor Constituents not Given)					
SiO_2_	Al_2_O_3_	Fe_2_O_3_	CaO	MgO	K_2_O
65.3	6.9	3.7	4.0	1.1	2.0
Physical properties					
Fineness—median particle size (µm)			5.1		
Specific gravity			1.8		
Blaine fineness (m^2^/kg)			900		

**Table 6:** Typical chemical and physical properties of bamboo leaf ash [63].

Major Chemical Composition (%) (Minor Constituents not Given)					
SiO_2_	Al_2_O_3_	Fe_2_O_3_	CaO	MgO	K_2_O
75.9	4.13	1.22	7.47	1.85	5.62
Physical properties					
Specific gravity			2.25		
Moisture content (%)			0.40		
